# Regulation of NAMPT in Human Gingival Fibroblasts and Biopsies

**DOI:** 10.1155/2014/912821

**Published:** 2014-02-25

**Authors:** Anna Damanaki, Marjan Nokhbehsaim, Sigrun Eick, Werner Götz, Jochen Winter, Gerhard Wahl, Andreas Jäger, Søren Jepsen, James Deschner

**Affiliations:** ^1^Clinical Research Unit 208, Center of Dento-Maxillo-Facial Medicine, University of Bonn, Welschnonnenstrasse 17, 53111 Bonn, Germany; ^2^Experimental Dento-Maxillo-Facial Medicine, Center of Dento-Maxillo-Facial Medicine, University of Bonn, 53111 Bonn, Germany; ^3^Department of Oral Surgery, Center of Dento-Maxillo-Facial Medicine, University of Bonn, 53111 Bonn, Germany; ^4^Department of Periodontology, Laboratory of Oral Microbiology, University of Bern, 3010 Bern, Switzerland; ^5^Department of Orthodontics, Center of Dento-Maxillo-Facial Medicine, University of Bonn, 53111 Bonn, Germany; ^6^Department of Periodontology, Operative and Preventive Dentistry, Center of Dento-Maxillo-Facial Medicine, University of Bonn, 53111 Bonn, Germany

## Abstract

Adipokines, such as nicotinamide phosphoribosyltransferase (NAMPT), are molecules, which are produced in adipose tissue. Recent studies suggest that NAMPT might also be produced in the tooth-supporting tissues, that is, periodontium, which also includes the gingiva. The aim of this study was to examine if and under what conditions NAMPT is produced in gingival fibroblasts and biopsies from healthy and inflamed gingiva. Gingival fibroblasts produced constitutively NAMPT, and this synthesis was significantly increased by interleukin-1**β** and the oral bacteria *P. gingivalis* and *F. nucleatum*. Inhibition of the MEK1/2 and NF**κ**B pathways abrogated the stimulatory effects of *F. nucleatum* on NAMPT. Furthermore, the expression and protein levels of NAMPT were significantly enhanced in gingival biopsies from patients with periodontitis, a chronic inflammatory infectious disease of the periodontium, as compared to gingiva from periodontally healthy individuals. In summary, the present study provides original evidence that gingival fibroblasts produce NAMPT and that this synthesis is increased under inflammatory and infectious conditions. Local synthesis of NAMPT in the inflamed gingiva may contribute to the enhanced gingival and serum levels of NAMPT, as observed in periodontitis patients. Moreover, local production of NAMPT by gingival fibroblasts may represent a possible mechanism whereby periodontitis may impact on systemic diseases.

## 1. Introduction

Periodontitis is a chronic inflammatory disease, which is characterized by the irreversible destruction of the tooth-supporting tissues, that is, periodontium. The periodontium consists of the gingiva, periodontal ligament (PDL), root cementum, and alveolar bone. Periodontopathogens, such as *Porphyromonas gingivalis* and *Fusobacterium nucleatum*, in the subgingival dental plaque are essential for the initiation and progression of periodontitis [1, 2]. However, cofactors, such as smoking or genetic predisposition, are also critical and represent established risk factors of periodontitis [3]. The periodontopathogens and their components and products can elicit an inflammatory host response, which involves inflammatory mediators, such as interleukin (IL)-1*β*, in the periodontal tissues. The inflammation, if exaggerated, can lead to matrix degradation and bone resorption and, thereby, formation of periodontal pockets. If periodontitis remains untreated, the disease can finally result in tooth loss [3, 4]. Periodontitis is one of the most frequent diseases worldwide and can have a tremendous impact on the physical, psychological, and social aspects of life [5, 6].

Interestingly, several meta-analyses have revealed that periodontitis is associated with obesity, diabetes type II, and metabolic syndrome [7–10]. Even though the underlying mechanisms for these associations are yet to be determined, it has been speculated that adipokines might represent an important pathomechanistic link between periodontitis and the aforementioned systemic diseases [11]. Adipokines, such as nicotinamide phosphoribosyltransferase (NAMPT), leptin, resistin, and adiponectin, are molecules, which are produced in adipose tissue [12]. However, there is increasing evidence that these adipokines are also synthesized in nonadipose tissues. In addition to their role in metabolic regulation, adipokines also modulate inflammatory and wound healing processes. Whereas NAMPT, leptin, and resistin have been shown to exert proinflammatory effects, adiponectin seems to be anti-inflammatory under most circumstances [13]. It has been suggested that the increased serum levels of proinflammatory adipokines, as found in a number of systemic diseases, could make affected individuals more susceptible to periodontitis [14–17].

NAMPT is mainly synthesized by macrophages and adipocytes in the adipose tissue and stimulates activation of nuclear factor-kappaB (NF*κ*B) as well as production of inflammatory molecules [18]. In obesity, metabolic syndrome, type 2 diabetes, and atherosclerosis, serum levels of NAMPT are increased [14–17]. Interestingly, NAMPT has also been found in gingival crevicular fluid (GCF) [19–21]. Even more importantly, the levels of NAMPT in GCF were significantly increased at inflamed sites, suggesting that NAMPT is also produced locally in the periodontium [19–21]. Recently, we have found that NAMPT is synthesized in PDL cells and that the constitutive production of NAMPT in these cells can be upregulated by inflammatory and microbial signals [22, 23]. Furthermore, NAMPT induced the production of proinflammatory and matrix-degrading molecules and also interfered with the regenerative capacity of PDL cells [23, 24]. These studies suggested that PDL cells can contribute to the increased NAMPT levels in GCF in periodontitis. However, whether other periodontal cells, such as gingival fibroblasts, which are much more exposed to periodontopathogens than PDL cells, also produce this proinflammatory adipokine, is as yet unknown. The aim of the present study was therefore to examine if, and under what conditions, NAMPT is produced in human gingival fibroblasts (HGF) and biopsies from healthy and inflamed gingiva.

## 2. Materials and Methods

### 2.1. Culture and Treatment of Cells

HGF were obtained from healthy gingiva of 8 individuals (mean age: 21.0 ± 1.6 years, min–max: 16–31 years; gender: 6 male/2 female), who had to undergo wisdom teeth extraction in the Department of Oral Surgery of the University of Bonn. Written informed consent and approval of the Ethics Committee of the University of Bonn were obtained (#043/11). HGF were grown in Dulbecco's minimal essential medium (DMEM, Invitrogen, Karlsruhe, Germany) supplemented with 10% fetal bovine serum (FBS, Invitrogen), 100 units penicillin, and 100 *μ*g/mL streptomycin (Invitrogen) at 37°C in a humidified atmosphere of 5% CO_2_. Cells from passages 3 to 5 were seeded (50,000 cells/well) on cell culture plates and grown to 80% confluence. One day prior to the experiment, the FBS concentration was reduced to 1%. In order to simulate inflammatory conditions in vitro, HGF were exposed to IL-1*β* (0.2–5 ng/mL; Calbiochem, San Diego, CA, USA), as done in our previous studies [25–27]. In order to mimic an infectious environment in vitro, HGF were incubated with the inactivated oral periodontopathogens *Porphyromonas gingivalis* ATCC 33277 and *Fusobacterium nucleatum* ATCC 25586 (optical density: 0.025, 0.05, and 0.1). Bacteria were suspended in PBS (OD_660 nm_ = 1, equivalent to 1.2 × 10^9^ bacterial cells/mL) and exposed two times to ultrasonication (160 W for 15 min) resulting in a complete killing, as previously reported [22, 23]. In some experiments, cells were also pre-incubated with specific inhibitors against NF*κ*B (pyrrolidine dithiocarbamate, PDTC; 10 *μ*M; Calbiochem) and MEK1/2 (U0126; 10 *μ*M; Calbiochem) signaling pathways 1 h prior to experiments.

### 2.2. Gingival Biopsies

Human gingiva was obtained from 10 periodontally healthy donors (mean age: 23.6 ± 1.7 years, min–max: 18–36 years; gender: 3 male/7 female), 10 gingivitis subjects (mean age: 31.4 ± 4.7 years, min–max: 17–66 years; gender: 7 male/3 female), and 10 periodontitis patients (mean age: 56.7 ± 5.5 years, min–max: 29–81 years; gender: 7 male/3 female). Exclusion criteria were presence of systemic diseases or medications as well as smoking. Sites were categorized into three groups according to the gingival index (GI), probing pocket depth (PD), clinical attachment level (CAL), and radiographic bone loss. Periodontally healthy sites were characterized by GI = 0 (no clinical inflammation), PD ≤ 3 mm, no CAL, and no radiographic bone loss. Sites with gingivitis had a GI > 1 (clinical inflammation), but also no periodontal pockets, attachment loss, and radiographic bone loss. Sites of periodontitis had also a GI > 1 and, additionally, PD ≥ 5 mm, CAL ≥ 3 mm, and radiographic bone loss. Gingiva was harvested during wisdom teeth removals or teeth extractions for orthodontic or periodontal reasons in the Department of Oral Surgery of the University of Bonn. Written informed consent and approval of the Ethics Committee of the University of Bonn were obtained (#043/11).

### 2.3. Real-Time PCR

RNA was extracted with an RNA extraction kit (Qiagen, Hilden, Germany). A total of 1 *μ*g of RNA was reverse transcribed using iScriptTM Select cDNA Synthesis Kit (Bio-Rad Laboratories, Munich, Germany) at 42°C for 90 min followed by 85°C for 5 min. The expression of NAMPT, adiponectin (Adipo) and its receptors (AdipoR1 and AdipoR2), leptin and its functional receptor (LeptinR), resistin, and glyceraldehyde-3-phosphate dehydrogenase (GAPDH) was detected by real-time PCR using the iCycler iQ detection system (Bio-Rad Laboratories), SYBR Green (Bio-Rad Laboratories), and specific primers (QuantiTect Primer Assay, Qiagen). One *μ*L of cDNA was amplified as a template in a 25 *μ*L reaction mixture containing 12.5 *μ*L 2x QuantiFast SYBR Green PCR Master Mix (Qiagen), 2.5 *μ*L of primers, and deionized water. The mixture was heated initially at 95°C for 5 min and then followed by 40 cycles with denaturation at 95°C for 10 s and combined annealing/extension at 60°C for 30 s. GAPDH was used as an endogenous control. The data were analyzed by the comparative threshold cycle method.

### 2.4. ELISA

The levels of NAMPT in the supernatants of HGF were determined by a commercially available ELISA kit (RayBiotech, Norcross, GA, USA) according to the manufacturer's instructions. The absorbance was measured with a microplate reader (PowerWave X, BioTek Instruments, Winooski, VT, USA) at 450 nm. The data were normalized by the cell number measured with an automatic cell counter (Moelab, Hilden, Germany).

### 2.5. Immunocytochemistry

HGF were grown in the presence and absence of IL-1*β*, *P. gingivalis*, or *F. nucleatum* on glass coverslips (Carl Roth, Karlsruhe, Germany) in 24-well plates for 48 h. Afterwards, cells were fixed in 4% paraformaldehyde (Sigma-Aldrich, Munich, Germany) at pH 7.4 and room temperature (RT) for 10 min and then permeabilized in 0.1% Triton X-100 (Sigma-Aldrich) for 5 min. Nonspecific antigens were blocked by incubation with serum block (Dako, Hamburg, Germany) for 20 min. Cells were then incubated with rabbit polyclonal antibody to NAMPT (Santa Cruz Biotechnology, Santa Cruz, CA, USA; 1 : 50) at 4°C overnight. Subsequently, cells were labeled with goat anti-rabbit IgG-HRP secondary antibody (Dako) for 30 min. For staining, cells were exposed to DAB chromogen (Thermo Fisher Scientific, Waltham, MA, USA) for 10 min at RT in the dark. After each incubation step, cells were washed twice with PBS (Invitrogen). Counterstaining was performed with Mayer's Hematoxylin (Merck Eurolab, Dietikon, Switzerland) for 1 min. Coverslips were mounted in Aquatex mounting agent (Merck Eurolab). Standardized photomicrographs were taken using an Axioskop 2 microscope (Carl Zeiss, Jena, Germany). The images were captured with an AxioCam MRc camera (Carl Zeiss) and the AxioVision 4.7 software (Carl Zeiss).

### 2.6. H&E Staining and Immunohistochemistry

Gingival biopsies were fixed in 4% paraformaldehyde (Sigma-Aldrich) for 2 days. Subsequently, the tissues were hydrated, then dehydrated in an ascending ethanol series (AppliChem, Darmstadt, Germany), and finally embedded in paraffin (McCormick Scientific, Richmond, IL, USA). Tissue sections of 2.5 *μ*m thickness were obtained, mounted on glass slides (Carl Roth), and dried at 37°C overnight. Sections from healthy and inflamed gingival tissues were stained with hematoxylin and eosin (H&E; Merck Eurolab). Selected tissue sections were then deparaffinized, rehydrated, and rinsed in PBS (Invitrogen) for 2 min. Endogenous peroxidase was blocked in 0.3% methanol (AppliChem)/H_2_O_2_ (Merck Eurolab) solution for 5 min in the dark. After rinsing, sections were pretreated with serum block (Dako) for 20 min. Then, sections were incubated with rabbit polyclonal antibody to NAMPT (Santa Cruz Biotechnology, 1 : 200) in a humid chamber at 4°C overnight. For detection of the antibody binding, sections were washed and then incubated with goat anti-rabbit IgG-HRP secondary antibody (Dako) at RT for 30 min. After rinsing, peroxidase activity was visualized with DAB chromogen (Thermo Fisher Scientific). Subsequently, all slides were rinsed and then counterstained with Mayer's hematoxylin (Merck Eurolab) for 1 min, dehydrated, and coverslipped for light microscopical analysis. Standardized photomicrographs were taken using an Axioskop 2 microscope (Carl Zeiss). The images were captured with an AxioCam MRc camera and the AxioVision 4.7 software (Carl Zeiss).

### 2.7. Statistical Analysis

For statistical analysis, the IBM SPSS Statistics 20 software was used. Mean values and standard errors of the mean (SEM) were calculated. All experiments were performed in triplicate and repeated at least twice. Parametric (ANOVA followed by the post hoc Dunnett test) and nonparametric (Mann-Whitney *U*) tests were applied. Differences between groups were considered significant at *P* < 0.05.

## 3. Results

### 3.1. Regulation of NAMPT mRNA Expression in HGF

**Figure 1:**
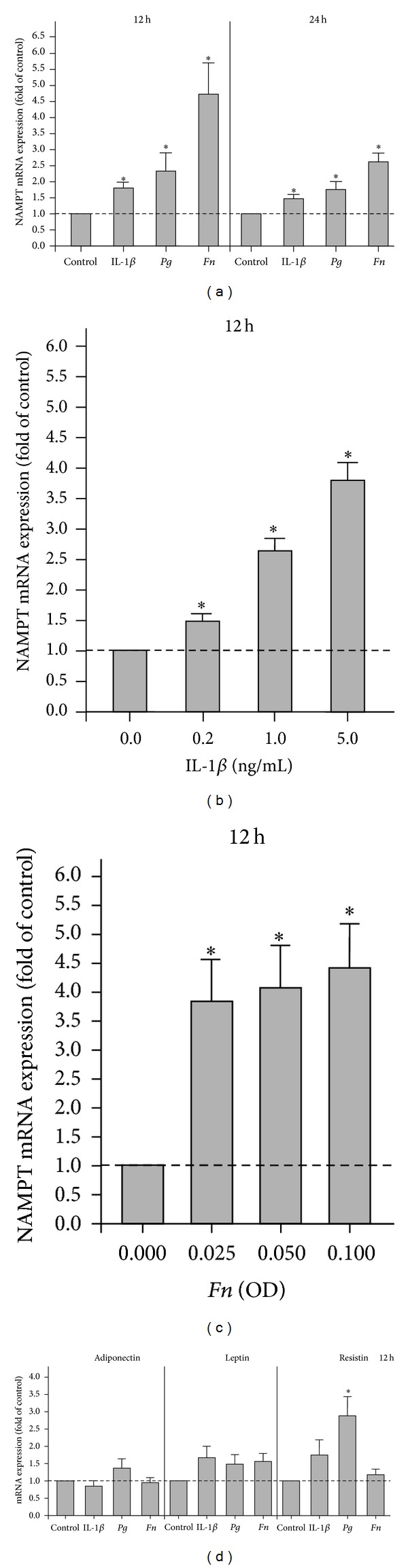
Expression of NAMPT and other adipokines in HGF. (a) Upregulation of NAMPT mRNA expression by IL-1*β*, *P. gingivalis* (*Pg*), and *F. nucleatum *(*Fn*) in HGF from 8 donors at 12 h and 24 h. (b) Stimulation of NAMPT mRNA expression by various concentrations (0.2, 1.0, and 5.0 ng/mL) of IL-1*β* in HGF from 3 donors at 12 h. (c) Stimulation of NAMPT mRNA expression by various doses (OD: 0.025, 0.050, and 0.100) of *F. nucleatum* in HGF from 3 donors at 12 h. (d) Expression of adiponectin, leptin, and resistin in IL-1*β*-, *P. gingivalis-* (*Pg*-), and *F. nucleatum- *(*Fn*-) treated HGF from 6 donors. Mean ± SEM; *significantly (*P* < 0.05) different from control.

First, we sought to examine whether HGF express NAMPT and, if so, whether the constitutive expression of NAMPT is modulated by inflammatory or microbial signals. As shown in Figure 1(a), HGF expressed spontaneously NAMPT and this expression was significantly enhanced by IL-1*β*, *P. gingivalis*, and *F. nucleatum* at 12 and 24 h. Further experiments revealed that the stimulatory effect of IL-1*β* on the NAMPT expression was dose-dependent, that is, the strongest upregulation of NAMPT was observed at the highest concentration of IL-1*β* (Figure 1(b)). By contrast, only a slight dose-dependency was found for the stimulatory action of *F. nucleatum* (Figure 1(c)) and no dose-dependency was observed for the effect of *P. gingivalis* (data not shown) on NAMPT. Preincubation of HGF with specific inhibitors against MEK1/2 and NF-*κ*B signaling reduced the *F. nucleatum*-induced upregulation of NAMPT by 64% and 83%, respectively.

### 3.2. Regulation of Adiponectin, Leptin, and Resistin mRNA Expressions in HGF

We also sought to study whether HGF produce additional adipokines and, if so, whether their expression can be regulated by IL-1*β*, *P. gingivalis*, and *F. nucleatum.* Our experiments demonstrated that HGF also express constitutively adiponectin, leptin, and resistin (Figure 1(d)). However, the constitutive expression of adiponectin was almost 200-fold and the constitutive expression of leptin and resistin was even 1000-fold less than that of NAMPT (data not shown). In general, IL-1*β*, *P. gingivalis*, and *F. nucleatum* had no significant effects on these adipokines except for the *P. gingivalis*-induced upregulation of resistin at 12 h (Figure 1(d)).

### 3.3. Regulation of NAMPT Protein Synthesis in HGF

**Figure 2:**
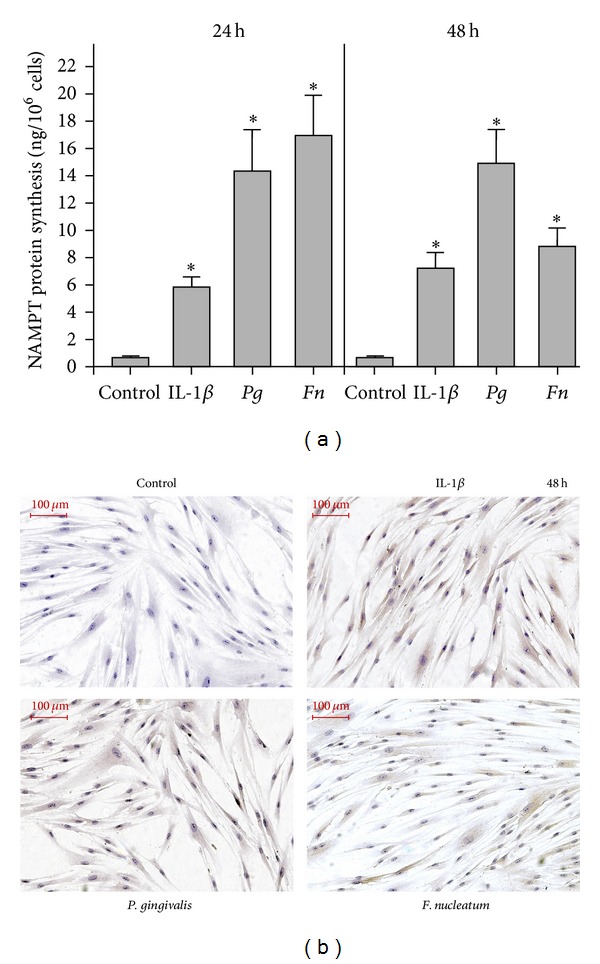
NAMPT protein levels in HGF. (a) NAMPT protein levels in supernatants of IL-1*β*-, *P. gingivalis-* (*Pg-*), and *F. nucleatum- *(*Fn-*) stimulated HGF from 3 donors at 24 h and 48 h. Mean ± SEM; *significantly (*P* < 0.05) different from control. (b) NAMPT protein in HGF in the presence and absence of IL-1*β*, *P. gingivalis,* and *F. nucleatum *at 48 h, as analyzed by immunocytochemistry. Images from one representative donor are shown.

The significant upregulation of NAMPT expression by IL-1*β*, *P. gingivalis*, and *F. nucleatum* was paralleled by increased NAMPT protein levels in the supernatants of stimulated cells, as compared to control cells, at 24 h and 48 h (Figure 2(a) and Table 1). Enhanced protein levels of NAMPT were also found in IL-1*β*- and *F. nucleatum*-stimulated cells by immunocytochemistry at 48 h (Figure 2(b)). By contrast, immunostaining for NAMPT was only slightly increased in* P. gingivalis*-treated cells at this time point (Figure 2(b)).

### 3.4. Expression of NAMPT and Other Adipokines in Gingival Biopsies

**Figure 3:**
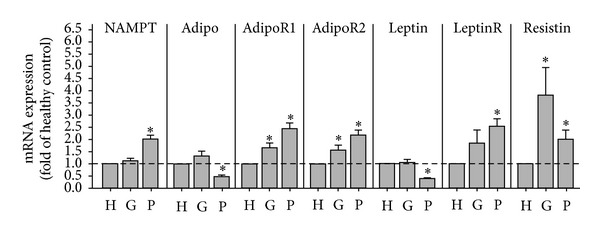
Expression of NAMPT and other adipokines in gingival biopsies. Expression of NAMPT, adiponectin (Adipo) and its receptors (AdipoR1 and AdipoR2), leptin and its receptor (LeptinR), and resistin in gingival biopsies from 10 periodontally healthy donors, 10 gingivitis subjects, and 10 periodontitis patients. H: healthy donors; G: gingivitis subjects; P: periodontitis patients. Mean ± SEM; *significantly (*P* < 0.05) different from periodontally healthy donors.

Next we studied the expression of NAMPT as well as other adipokines and their receptors in gingival biopsies from periodontally healthy, gingivitis, and periodontitis subjects. As demonstrated in Figure 3, the expression of NAMPT was significantly enhanced in gingiva from periodontitis patients as compared to gingiva from periodontally healthy subjects. Moreover, the expression of resistin was significantly increased in gingivitis and periodontitis. In contrast to NAMPT and resistin, leptin and adiponectin expressions were significantly reduced in gingiva from periodontitis patients. Interestingly, the downregulation of leptin and adiponectin was paralleled by a significant upregulation of their receptors in gingival biopsies from periodontitis subjects (Figure 3).

### 3.5. NAMPT Protein in Gingival Biopsies

**Figure 4:**
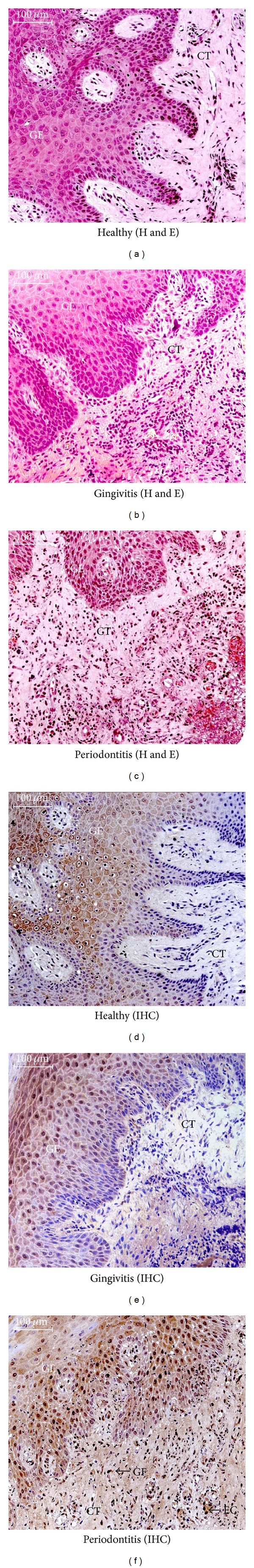
NAMPT protein levels in gingival biopsies. Gingiva from periodontally healthy ((a) and (d)), gingivitis ((b) and (e)), and periodontitis ((c) and (f)) subjects. Hematoxylin and eosin stain ((a)–(c)). NAMPT protein, as demonstrated by immunohistochemistry ((d)–(f)). Images from one representative individual of each group are shown. GE: gingival epithelium; CT: connective tissue, GF: fibroblast; EC: endothelial cell.

As shown in Figures 4(a)–4(c), healthy gingiva showed only few immunoinflammatory cells, whereas a pronounced round cell infiltrate was observed in biopsies from gingivitis and periodontitis subjects, as analyzed by hematoxylin and eosin staining. In healthy gingiva, NAMPT was mainly localized in the upper and intermediate layers of the epithelium, as evidenced by immunohistochemistry (Figure 4(d)). Interestingly, no immunolabeling was found in the epithelial basal layer and in the subepithelial connective tissue. Similar findings were observed in biopsies from gingivitis subjects. However, some weak immunolabeling was additionally present in the subepithelial connective tissue (Figure 4(e)). In gingiva from periodontitis patients, NAMPT was diffusely distributed in all layers of the epithelium (Figure 4(f)). Moreover, NAMPT was also observed in the subepithelial connective tissue, where immunostaining was found in the cytoplasm of fibroblasts and endothelial cells as well as in the extracellular space (Figure 4(f)).

## 4. Discussion

The present study provides original evidence that HGF produce NAMPT, and this synthesis is stimulated by the proinflammatory cytokine IL-1*β* and the periodontopathogens *P. gingivalis* and *F. nucleatum*. These findings suggest that local synthesis of NAMPT in the inflamed gingiva may contribute to the enhanced gingival and serum levels of NAMPT, as observed in patients with periodontitis. The local production of NAMPT by periodontal cells may represent a possible mechanism whereby periodontitis may impact on systemic diseases, such as diabetes mellitus and cardiovascular diseases. Moreover, since NAMPT stimulates the synthesis of proinflammatory and matrix-degrading molecules, increased NAMPT production by HGF at inflamed sites may result in the amplification of periodontal inflammation and destruction.

In a subset of experiments, we also studied the expression of adiponectin, leptin, and resistin in HGF. Although these adipokines could also be detected by real-time PCR, their expressions were very slight and barely modulated. A few studies have reported that HGF express AdipoR1 and AdipoR2, but not adiponectin, which seems to be in contrast to our findings [28, 29]. However, in these studies, the expression of adiponectin was analyzed by end-point PCR and immunoblotting, but not real-time PCR, which is a highly sensitive and quantitative assay. In our study, the constitutive expression of adiponectin was almost 200-fold less than that of NAMPT. Therefore, the aforementioned studies may well concur with our observation that production of adiponectin by HGF is negligible. In one study, the presence of leptin and its receptor in gingival tissue was studied by immunohistochemistry [30]. Leptin and its receptor were found in epithelial, endothelial, and inflammatory cells, but not subepithelial fibroblasts. In the present study, the constitutive expression of leptin was 1000-fold less than the expression of NAMPT. Therefore, the observations by Ay et al. [30] may also be in line with our findings that leptin production by HGF may not be significant. As far as we know, there are no reports about the presence of resistin in HGF so far.

In the present study, HGF were exposed to IL-1*β*, which was used to mimic inflammatory conditions in vitro, as in our previous studies [25–27]. This proinflammatory cytokine has been shown to be increased in GCF and gingival tissues at inflamed sites [31–33]. In order to simulate a microbial environment in vitro, HGF were treated with a suspension of *P. gingivalis* and *F. nucleatum*. The suspensions were exposed to intensive ultrasonication and contained disrupted cell wall particles with a high amount of lipopolysaccharide. Nevertheless, other microbial components may also have been present in the suspensions.* P. gingivalis*, which is a gram-negative bacterium and strongly linked to periodontitis, possesses various virulence factors, such as gingipains and fimbriae. This bacterium has been shown to invade host cells and evade the host defense system [34–36]. *F. nucleatum* is also a gram-negative, anaerobic microorganism. This bacterium acts as a bridge bacterium between early and late colonizers during plaque development and is associated with both gingivitis and periodontitis. Like *P. gingivalis*, *F. nucleatum* has also been demonstrated to invade host cells [37–42]. Nonetheless, periodontal diseases are caused by a complex bacterial biofilm and further studies should clarify whether other microorganisms, which are associated with periodontitis, also stimulate the synthesis of NAMPT in HGF. In order to ensure that data were comparable, IL-1*β*, *P. gingivalis,* and *F. nucleatum* were applied at the same doses as in previous studies [22, 23].

Our findings for NAMPT at transcriptional levels were paralleled by the findings obtained by ELISA. IL-1*β* and the periodontopathogens *P. gingivalis* and *F. nucleatum* caused increased NAMPT levels in the supernatants of the stimulated cells. In general, the ELISA results were also in agreement with findings from the immunocytochemistry analysis, which demonstrated increased NAMPT protein levels in IL-1*β*- and *F. nucleatum*-stimulated cells, as compared to control. However, in contrast to the ELISA data, only slight differences in NAMPT protein levels were found between *P. gingivalis*-treated and control cells by immunocytochemistry. Whether *P. gingivalis* leads to a fast and almost complete secretion of NAMPT in HGF or whether other mechanisms play a role needs to be determined in further studies.

We also sought to unravel the intracellular mechanisms underlying the upregulation of NAMPT in HGF. Our experiments revealed that the *F. nucleatum*-induced NAMPT upregulation was dependent on MEK1/2 and NF*κ*B signaling. These results are in line with our previous findings on PDL cells, in which the NF*κ*B pathway was also activated by *F. nucleatum* [22]. Further studies should examine what pathways are used by IL-1*β* and *P. gingivalis* for their stimulatory effects on NAMPT expression.

In order to verify the results from our in vitro experiments, in which inflammatory and infectious conditions were simulated, we also studied the expression and protein levels of NAMPT in gingival biopsies from periodontally healthy, gingivitis, and periodontitis subjects. To our knowledge, this is the first study which demonstrates that the gingival expression of NAMPT is increased at sites of periodontitis as compared to healthy sites. This finding is in accordance with our in vitro results, which revealed that NAMPT expression and protein levels are increased under inflammatory and infectious conditions. However, gingival biopsies contain various cell types, such as fibroblasts, epithelial, endothelial, and inflammatory cells, and each of them might be a possible source of NAMPT. We therefore also studied the production of NAMPT in gingival tissue by immunohistochemistry. Whereas no or only very low levels of NAMPT were found in the subepithelial connective tissue of healthy gingival biopsies, high amounts of NAMPT were observed in the connective tissue of gingiva from periodontitis patients. Immunostaining for NAMPT was detected in the cytoplasm of fibroblasts and endothelial cells as well as in the extracellular space. Interestingly, NAMPT was also present in epithelial cells in both healthy and diseased tissues. These results confirm our in vitro data on the increased NAMPT production in gingival fibroblasts under inflammatory and infectious conditions. Furthermore, our data provide first evidence that gingival epithelial cells produce NAMPT.

We also sought to examine the expression of other adipokines in gingival biopsies. Our study shows for the first time that the proinflammatory adipokine resistin is also significantly upregulated in inflamed gingival tissue as compared to healthy gingiva. By contrast, the leptin and adiponectin expressions were decreased in gingiva from periodontitis patients. The downregulation of these two adipokines was paralleled by the upregulation of their receptors in periodontitis. In a recent study, the expression of both adiponectin receptors was lower in periodontal tissues from patients with severe periodontitis than in healthy periodontal tissues [43]. It was therefore speculated that adiponectin may not function efficiently at sites of periodontitis due to the decrease in the number of its receptors. However, this study only included a small number of individuals. Furthermore, immunoblotting was used in contrast to our study, where real-time PCR analysis was performed. One study focused on leptin protein levels in gingiva from healthy sites and sites of gingivitis or periodontitis [44]. This study showed a significant decrease in the concentration of gingival leptin, as analyzed by ELISA, and therefore concurs with our finding at transcriptional level. Our findings are also supported by another study, which demonstrated that leptin is present within healthy and inflamed gingiva and decreases in concentration as the adjacent probing depth increases [45]. A third study, in which an immunohistochemistry analysis was performed, also demonstrated the presence of leptin and its receptor in gingival tissue. However, no differences in tissue staining distribution and intensity of leptin and its receptor between healthy and inflamed periodontal conditions were found, which might be due to the less sensitive assay used in this study [30].

In summary, the present study provides original evidence that gingival fibroblasts produce NAMPT and that this synthesis is increased under inflammatory and infectious conditions. Local synthesis of NAMPT in the inflamed gingiva may contribute to the enhanced gingival and serum levels of NAMPT, as observed in periodontitis patients. Moreover, local production of NAMPT by gingival fibroblasts may represent a possible mechanism whereby periodontitis may impact on systemic diseases.

## Figures and Tables

**Table 1:** Regulation of NAMPT protein levels, as analyzed by ELISA.

Group	NAMPT (ng/10^6^ cells)	
	24 h	48 h
Control	0.66 ± 0.10	0.65 ± 0.07
IL-1*β*	5.85 ± 0.77*	7.27 ± 1.09*
*P. gingivalis *	14.35 ± 3.01*	14.99 ± 2.37*
*F. nucleatum *	17.00 ± 2.87*	8.86 ± 1.28*

NAMPT protein levels in supernatants from IL-1*β*-, *P. gingivalis*-, and *F. nucleatum*-stimulated HGF (6 donors) at 24 h and 48 h. Untreated cells served as control. Mean ± SEM; *significantly (*P* < 0.05) different from control.
